# Timing of Intraoperative Dexmedetomidine Administration and Post-anaesthesia Care Unit Recovery: A Single-Centre Quality Improvement Audit

**DOI:** 10.7759/cureus.112424

**Published:** 2026-07-10

**Authors:** Ayani Gamage, Lucie Voldanova, Samhita Gutta

**Affiliations:** 1 Anaesthesia, Royal Brisbane and Women's Hospital, Brisbane, AUS

**Keywords:** dexmedetomidine, pacu, postoperative complications, prolonged intubation, recovery

## Abstract

Background

Dexmedetomidine is increasingly used in multimodal perioperative analgesia due to its sedative, anxiolytic, and opioid-sparing properties. However, concerns remain regarding residual sedation, haemodynamic instability, and delayed post-anaesthesia care unit (PACU) discharge, particularly when administered close to the end of surgery. This audit aimed to characterise intraoperative dexmedetomidine use at a tertiary Australian hospital and evaluate the relationship between administration timing, PACU clinical recovery time, and postoperative complications.

Methods

A retrospective single-centre quality improvement audit was conducted at the Royal Brisbane and Women’s Hospital, Brisbane, Queensland, Australia. Adult surgical patients who received intraoperative dexmedetomidine between 1 January 2025 and 19 February 2025 were identified from electronic anaesthetic records. Patients were excluded if they were younger than 18 years, were admitted directly to intensive care, or did not recover in PACU. Data collected included anaesthetic technique, dexmedetomidine dose and administration method, timing of administration, PACU clinical recovery time, sedation scores, pain scores, oral morphine equivalent requirements and postoperative complications. Categorical outcomes were compared using Fisher’s exact test, while non-parametric continuous outcomes were compared using the Mann-Whitney U test.

Results

Of 230 records reviewed, 225 patients met the inclusion criteria. General anaesthesia was used in 162 (72.0%) cases, while regional anaesthesia or procedural sedation was used in 63 (28.0%). Dexmedetomidine was most commonly administered as a bolus only, occurring in 188 (83.6%) patients. Median PACU clinical recovery time was 64 minutes. Postoperative opioid requirements were low, with seven (3.1%) patients requiring more than 60 mg of oral morphine equivalents. Overall, bradycardia occurred in 53 (23.6%) patients, hypotension in 24 (10.7%), and postoperative nausea and vomiting in 20 (8.9%). Dexmedetomidine administration within 60 minutes of procedure completion was associated with shorter median PACU clinical recovery time than administration more than 60 minutes before completion (58.5 vs 83.0 minutes, Mann-Whitney U=4418, p=0.006). Bradycardia, hypotension, and postoperative nausea and vomiting were numerically more common in the within-60-minute group, although these differences were not statistically significant.

Conclusion

PACU recovery outcomes were generally favourable in this cohort of adult surgical patients who received intraoperative dexmedetomidine, with low postoperative opioid requirements. Administration within 60 minutes of procedure completion was associated with shorter median PACU clinical recovery time than earlier administration; however, this retrospective observational audit cannot determine causality, and the finding may reflect confounding by procedure duration, anaesthetic technique, dose, surgical complexity or case mix. Bradycardia, hypotension, and postoperative nausea and vomiting were numerically more common with later administration, but these differences were not statistically significant. These findings should therefore be interpreted as exploratory and hypothesis-generating, with prospective studies and adjusted analyses required to determine whether dexmedetomidine timing independently influences PACU recovery or early postoperative complications.

## Introduction

Dexmedetomidine is a highly selective alpha-2 adrenoceptor agonist that is increasingly used in contemporary anaesthetic practice as an adjunct to multimodal peri-operative analgesia and sedation [1,2]. Its clinical effects include sedation, anxiolysis, sympatholysis, and analgesic-sparing activity, while preserving respiratory drive to a greater extent than many traditional sedative agents [1,2]. These properties have made dexmedetomidine an attractive option across a wide range of anaesthetic contexts, including general anaesthesia, regional anaesthesia, procedural sedation and enhanced recovery pathways.

The increasing use of dexmedetomidine reflects a broader shift in perioperative medicine towards opioid-sparing and multimodal analgesic strategies. Opioid-sparing approaches aim to reduce opioid-related adverse effects, including respiratory depression, ileus, nausea, vomiting, sedation and delayed mobilisation. In this context, dexmedetomidine may provide useful perioperative benefits by reducing intraoperative anaesthetic requirements, improving haemodynamic responses to surgical stimulation and lowering postoperative opioid requirements. Perioperative guidelines and previous studies have reported opioid-sparing and recovery-related benefits, including reduced postoperative pain, reduced opioid consumption, reduced emergence agitation, and reduced postoperative nausea and vomiting [3-5].

Despite these potential benefits, the effect of dexmedetomidine on post-anaesthesia care unit (PACU) recovery remains uncertain. Its pharmacodynamic profile includes dose-dependent sympatholysis, which can result in bradycardia and hypotension [1,2]. In addition, its sedative effects may persist into the early postoperative period, raising concerns regarding delayed emergence, prolonged PACU observation, and delayed discharge readiness [1,2,6]. These concerns are particularly relevant in high-throughput operating theatre environments, where small increases in recovery time may have implications for patient flow, PACU capacity, and theatre efficiency.

The relationship between dexmedetomidine and postoperative recovery has been reported with mixed outcomes in the literature. While some studies found no significant association between the two [6], others reported a dose-dependent association between intraoperative dexmedetomidine and prolonged postoperative recovery [7]. A recent study also found that dexmedetomidine may be associated with prolonged PACU recovery in certain surgical populations [8]. These differing findings suggest that the impact of dexmedetomidine may depend not only on total dose and anaesthetic technique, but also on how and when it is administered during the procedure.

The timing of intraoperative dexmedetomidine administration is a clinically important but less clearly defined aspect of practice. Administration early in a procedure may allow the desired sedative and analgesic-sparing effects to occur intraoperatively while limiting residual effects during recovery. Conversely, administration closer to emergence may increase the likelihood that haemodynamic or sedative effects persist into PACU. At the Royal Brisbane and Women’s Hospital (RBWH), variation exists in both the dose and timing of dexmedetomidine administration. Anecdotal concerns from PACU and anaesthetic staff suggest that administration near the end of surgery may be associated with delayed recovery and increased postoperative complications.

Objectives

The objectives of this quality improvement audit were to: (i) describe current intraoperative dexmedetomidine administration practices at RBWH, (ii) evaluate the association between dexmedetomidine administration timing and PACU clinical recovery time, and (iii) assess early postoperative outcomes, including sedation, pain scores, postoperative opioid requirements, bradycardia, hypotension, and postoperative nausea and vomiting.

## Materials and methods

Study design and setting

This was a retrospective single-centre quality improvement audit conducted at the RBWH, a tertiary referral hospital in Brisbane, Queensland, Australia. The project was approved as a quality improvement activity by the Metro North Health (approval number EX/2025/MNH/123831). As the project was conducted as a retrospective audit of routinely collected clinical data, individual patient consent was not required.

Study population

Electronic anaesthetic records were reviewed for all adult surgical patients who received intraoperative dexmedetomidine between 1 January 2025 and 19 February 2025. Patients were eligible for inclusion if they were aged 18 years or older, received dexmedetomidine during the intraoperative period, and recovered in the PACU following surgery or procedural intervention. Patients were excluded if they were younger than 18 years of age, were admitted directly to the intensive care unit postoperatively, or did not have relevant PACU recovery data available for analysis.

Data collection

Data were extracted retrospectively from electronic anaesthetic and PACU records. PACU arrival time, documented discharge-readiness time, and postoperative observations were obtained from routine electronic PACU documentation. Discharge readiness was defined as the time at which the patient was documented as clinically suitable to leave PACU according to local PACU discharge practice, rather than the actual physical time of PACU departure. This approach was used to minimise the influence of non-clinical delays, including ward bed availability, transport delays and administrative factors.

Other collected variables included anaesthetic technique, procedure duration, dexmedetomidine dose, method of dexmedetomidine administration, timing of administration in relation to procedure completion, time to extubation where applicable, PACU sedation score, postoperative pain score, oral morphine equivalent requirements and documented postoperative complications. Complications of interest included bradycardia, hypotension, and postoperative nausea and vomiting.

Dexmedetomidine administration was categorised as bolus only, infusion only, or combined bolus and infusion. Anaesthetic technique was categorised as general anaesthesia or regional anaesthesia/procedural sedation. Procedures were stratified by duration into procedures lasting less than 60 minutes and those lasting 60 minutes or longer. A secondary timing analysis compared patients who received dexmedetomidine within 60 minutes of procedure completion with those who received dexmedetomidine more than 60 minutes before procedure completion.

Patients with unavailable PACU recovery data were excluded from the analysis. Where individual outcome data were unavailable, analyses were performed using available data only, with denominators reflecting the number of patients with recorded values.

Outcome measures

The primary outcome was PACU clinical recovery time, defined as the interval from arrival in PACU to the time the patient was clinically ready for PACU discharge. This reflected the achievement of discharge readiness based on local PACU clinical criteria and did not include non-clinical delays after discharge readiness had been achieved, such as delays related to ward bed availability, transport, or administrative factors.

Secondary outcomes included time to extubation in patients receiving general anaesthesia, PACU sedation score, postoperative pain score, postoperative oral morphine equivalent requirement, and early postoperative complications. Time to extubation was defined as the interval from the final intraoperative dexmedetomidine dose to documented extubation. PACU sedation score and pain score were extracted from PACU nursing observations, with pain assessed using a visual analogue scale from 0 to 10.

Postoperative opioid requirement was converted to oral morphine equivalents, with higher opioid requirement defined as greater than 60 mg oral morphine equivalent during the postoperative recovery period. Bradycardia was defined as a heart rate <60 beats/minute. Hypotension was defined as systolic blood pressure <90 mmHg, mean arterial pressure <65 mmHg, or documentation of hypotension requiring clinical intervention. Postoperative nausea and vomiting were defined as documented nausea or vomiting in the PACU or the administration of antiemetic therapy for suspected nausea or vomiting. 

These outcomes were selected because they reflect clinically relevant domains of early postoperative recovery, including discharge readiness, emergence from anaesthesia, sedation, analgesic requirement, haemodynamic stability and postoperative tolerance of anaesthesia.

Statistical analysis

Descriptive statistics were used to summarise patient characteristics, dexmedetomidine administration patterns and PACU outcomes. Continuous variables were reported as medians with interquartile ranges where data were non-normally distributed or means with standard deviations where appropriate. Categorical variables were reported as frequencies and percentages. Comparisons between categorical variables were performed using Fisher’s exact test because several subgroup event counts were small. For categorical outcomes analysed using Fisher’s exact test, odds ratios (ORs) are reported as the test statistic in the tables. Continuous non-parametric outcomes were compared using the Mann-Whitney U test, with the U statistic reported in the tables. A p-value of less than 0.05 was considered statistically significant. As this was a retrospective quality improvement audit, statistical analyses were interpreted as exploratory rather than confirmatory.

No multivariable adjustment was performed; therefore, comparisons were unadjusted, and potential confounding by patient comorbidities, intraoperative fluid therapy, concurrent medications, anaesthetic technique, procedure duration, surgical specialty, dexmedetomidine dose, and case complexity could not be controlled for.

## Results

Patient characteristics and dexmedetomidine administration patterns

A total of 230 patient records were reviewed. Five patients were excluded because they did not meet eligibility criteria, leaving 225 patients for final analysis. General anaesthesia accounted for 162 (72.0%) cases, while regional anaesthesia or procedural sedation accounted for 63 (28.0%) cases. Dexmedetomidine was most commonly administered as a bolus alone, occurring in 188 (83.6%) patients. Infusion-only administration occurred in 21 (9.3%) patients, while combined bolus and infusion administration occurred in 16 (7.1%) patients.

The median dexmedetomidine dose was higher in patients receiving general anaesthesia than in those receiving regional anaesthesia or procedural sedation. In the general anaesthesia group, the median dose was 0.33 mcg/kg, compared with 0.15 mcg/kg in the regional anaesthesia and procedural sedation group. This difference likely reflects differences in procedural complexity, anaesthetic goals and the use of dexmedetomidine as an adjunct to general anaesthesia rather than primarily as a sedative agent.

Overall PACU recovery outcomes

Overall PACU recovery outcomes were generally favourable. The median PACU clinical recovery time was 64 minutes, and the median sedation score was 2. The mean postoperative pain score was 3.4 with a standard deviation of 1.8. Postoperative opioid requirements were low, with seven (3.1%) patients requiring more than 60 mg of oral morphine equivalents postoperatively.

Postoperative complications were observed in a minority of patients. Bradycardia occurred in 53 (23.6%) patients, hypotension in 24 (10.7%) patients, and postoperative nausea and vomiting in 20 (8.9%) patients. These findings indicate that overall recovery profiles were acceptable within this cohort, although haemodynamic events remained clinically relevant.

General anaesthesia subgroup

Among the 162 patients who received general anaesthesia, 66 underwent procedures lasting <60 minutes and 96 underwent procedures lasting ≥60 minutes. Procedures lasting <60 minutes were associated with a shorter median PACU clinical recovery time than procedures lasting ≥60 minutes, with median recovery times of 77.5 minutes and 89.0 minutes, respectively. Median time from last dexmedetomidine dose to extubation was also shorter in the <60-minute group, at 49.0 minutes compared with 76.0 minutes in the ≥60-minute group. General anaesthesia subgroup outcomes are summarised in Table 1.

**Table 1:** General anaesthesia subgroup outcomes by procedure duration (N=162) Note: Continuous variables are presented as median (IQR) and were compared using the Mann-Whitney U test, with U reported as the test statistic. Categorical variables are presented as n (%) and were compared using Fisher’s exact test, with odds ratio (OR) reported as the test statistic. PACU: post-anaesthesia care unit; PONV: postoperative nausea and vomiting; VAS: visual analogue scale

Outcome	Procedures <60 minute (n=66)	Procedures ≥60 minutes (n=96)	Test statistic	p-value
PACU clinical recovery time (minutes), median (IQR)	77.5 (IQR 51.0-124.3)	89.0 (IQR 59.8-127.0)	U=2800	0.210
Time from last dose to extubation (minutes), median (IQR)	49.0 (IQR 38.3-73.8)	76.0 (IQR 22.3-146.8)	U=2078	0.037
Hypotension, n (%)	9 (13.6%)	9 (9.4%)	OR=1.53	0.450
Bradycardia, n (%)	18 (27.3%)	22 (22.9%)	OR=1.26	0.580
PONV, n (%)	5 (7.6%)	8 (8.3%)	OR=0.90	1.000
Sedation score 3, n (%)	12 (18.2%)	28 (29.2%)	OR=0.54	0.139
VAS >7, n (%)	12 (18.2%)	14 (14.6%)	OR=1.30	0.664

Hypotension occurred in nine (13.6%) patients undergoing procedures lasting <60 minutes and nine (9.4%) patients undergoing procedures lasting ≥60 minutes (p=0.450). Bradycardia occurred in 18 (27.3%) patients in the <60-minute group and 22 (22.9%) patients in the ≥60-minute group (p=0.580). Postoperative nausea and vomiting occurred in five (7.6%) patients in the <60-minute group and eight (8.3%) patients in the ≥60-minute group (p=1.000). Higher sedation scores were more common in the ≥60-minute procedure group. A sedation score of 3 was documented in 12 (18.2%) patients undergoing procedures lasting <60 minutes and 28 (29.2%) patients undergoing procedures lasting ≥60 minutes (p=0.139). Severe pain, defined as a visual analogue scale score >7, occurred in 12 (18.2%) patients in the <60-minute group and 14 (14.6%) patients in the ≥60-minute group (p=0.664).

Overall, these findings do not support the hypothesis that shorter procedures under general anaesthesia were associated with prolonged PACU recovery following dexmedetomidine administration.

Regional anaesthesia and sedation subgroup

Among the 63 patients who received regional anaesthesia or procedural sedation, 41 underwent procedures lasting <60 minutes and 22 underwent procedures lasting ≥60 minutes. As observed in the general anaesthesia subgroup, shorter procedures were associated with shorter PACU clinical recovery time. The median PACU clinical recovery time was 31.0 minutes in the <60-minute group compared with 34.5 minutes in the ≥60-minute group. Regional anaesthesia and sedation subgroup outcomes are summarised in Table 2.

**Table 2:** Regional sedation subgroup outcomes by procedure duration (N=63) Note: Continuous variables are presented as median (IQR) and were compared using the Mann-Whitney U test, with U reported as the test statistic. Categorical variables are presented as n (%) and were compared using Fisher’s exact test, with odds ratio (OR) reported as the test statistic. PACU: post-anaesthesia care unit; PONV: postoperative nausea and vomiting

Outcome	Procedures <60 minutess (n=41)	Procedures ≥60 minutes (n=22)	Test statistic	p-value
PACU clinical recovery time (minute), median (IQR)	31.0 (IQR 25.0-39.0)	34.5 (IQR 28.3-46.5)	U=359	0.187
Hypotension, n (%)	4 (9.8%)	1 (4.5%)	OR=2.27	0.650
Bradycardia, n (%)	9 (22.0%)	4 (18.2%)	OR=1.27	1.000
PONV, n (%)	6 (14.6%)	1 (4.5%)	OR=3.60	0.405
Sedation score ≥2, n (%)	2 (4.9%)	2 (9.1%)	OR=0.51	0.606

In this subgroup, hypotension occurred in four (9.8%) patients undergoing procedures lasting <60 minutes and one (4.5%) patient undergoing procedures lasting ≥60 minutes (p=0.650). Bradycardia occurred in 9 (22.0%) patients in the <60-minute group and 4 (18.2%) patients in the ≥60-minute group (p=1.000). Postoperative nausea and vomiting occurred in six (14.6%) patients in the <60-minute group and one (4.5%) patient in the ≥60-minute group (p=0.405). Sedation scores ≥2 occurred in two (4.9%) patients in the <60-minute group and two (9.1%) patients in the ≥60-minute group (p=0.606).

Although complication rates were numerically higher in shorter regional anaesthesia or sedation cases for some outcomes, these differences were not statistically significant. Interpretation is limited by the smaller size of this subgroup and the low number of events.

Timing of dexmedetomidine administration

Dexmedetomidine was administered within 60 minutes of procedure completion in 148 (65.8%) patients and more than 60 minutes before procedure completion in 77 (34.2%) patients. Patients who received dexmedetomidine within 60 minutes of procedure completion demonstrated a significantly shorter median PACU clinical recovery time than those who received dexmedetomidine earlier in the procedure (58.5 minutes vs 83.0 minutes, Mann-Whitney U=4418, p=0.006).

Despite the shorter PACU clinical recovery time observed in the within-60-minute group, postoperative complications were numerically more common. Bradycardia occurred in 37 (25.0%) patients who received dexmedetomidine within 60 minutes of procedure completion and 16 (20.8%) patients who received it earlier (p=0.512). Hypotension occurred in 18 (12.2%) and six (7.8%) patients, respectively (p=0.370). Postoperative nausea and vomiting occurred in 15 (10.1%) patients in the within-60-minute group and five (6.5%) patients in the earlier-administration group (p=0.463). None of these differences reached statistical significance. Timing-based outcomes are summarised in Table 3.

**Table 3:** Outcomes by timing of dexmedetomidine administration Note: Continuous variables are presented as median (IQR) and were compared using the Mann-Whitney U test, with U reported as the test statistic. Categorical variables are presented as n (%) and were compared using Fisher’s exact test, with odds ratio (OR) reported as the test statistic. PACU: post-anaesthesia care unit; PONV: postoperative nausea and vomiting

Outcome	Dexmedetomidine within 60 minutes (n=148)	Dexmedetomidine >60 minutes before completion (n=77)	Test statistic	p-value
PACU clinical recovery time (minutes), median (IQR)	58.5 (IQR 35.0-112.3)	83.0 (IQR 53.0-123.0)	U=4418	0.006
Bradycardia, n (%)	37 (25.0%)	16 (20.8%)	OR=1.27	0.512
Hypotension, n (%)	18 (12.2%)	6 (7.8%)	OR=1.64	0.370
PONV, n (%)	15 (10.1%)	5 (6.5%)	OR=1.62	0.463

## Discussion

This single-centre quality improvement audit evaluated intraoperative dexmedetomidine administration practices and early postoperative recovery outcomes in 225 adult surgical patients. Dexmedetomidine was most commonly administered as a bolus, overall PACU recovery outcomes were acceptable, and postoperative opioid requirements were low. Administration within 60 minutes of procedure completion was associated with significantly shorter PACU clinical recovery time compared with administration earlier in the procedure. However, bradycardia, hypotension and postoperative nausea and vomiting were numerically more common in the within-60-minute group, although none of these differences reached statistical significance.

These findings sit within a mixed body of literature regarding dexmedetomidine and postoperative recovery. Sin et al. reported that dexmedetomidine did not significantly increase PACU length of stay, although it was associated with increased time to extubation and a higher incidence of hypotension [6]. In contrast, Ma et al. reported a dose-dependent association between intraoperative dexmedetomidine and prolonged recovery, particularly in shorter procedures and monitored anaesthesia care cases [7]. More recent retrospective cohort data have similarly suggested that dexmedetomidine exposure may be associated with prolonged PACU recovery in selected surgical populations [8]. In the present audit, shorter procedures were not associated with prolonged PACU clinical recovery time in either the general anaesthesia or regional anaesthesia/procedural sedation subgroups.

The finding that dexmedetomidine administration within 60 minutes of procedure completion was associated with shorter PACU clinical recovery time contrasts with the initial hypothesis that administration closer to emergence may prolong recovery through residual sedative or haemodynamic effects. However, this association should be interpreted cautiously. Patients who received dexmedetomidine earlier in the procedure may have undergone longer or more complex procedures, or differed in anaesthetic technique, surgical specialty, dexmedetomidine dose or case mix. As this audit was retrospective, did not include a non-dexmedetomidine control group, and did not perform multivariable adjustment, the observed association should be regarded as exploratory and hypothesis-generating. It should not be interpreted as evidence that later dexmedetomidine administration independently shortens PACU recovery.

Although later administration was associated with shorter PACU clinical recovery time, postoperative complications were numerically more common in this group. This pattern is biologically plausible given the known pharmacodynamic effects of dexmedetomidine, including sympatholysis, bradycardia, hypotension, and sedation [1,2]. However, the differences in bradycardia, hypotension, and postoperative nausea and vomiting did not reach statistical significance. These findings are therefore best regarded as hypothesis-generating rather than confirmatory.

The low postoperative opioid requirement observed in this cohort is consistent with the use of dexmedetomidine as part of opioid-sparing perioperative practice. Only seven (3.1%) patients required more than 60 mg of oral morphine equivalents postoperatively. This is consistent with perioperative analgesia guidance and previous studies describing reduced postoperative opioid consumption and analgesic-sparing effects with alpha-2 agonists and dexmedetomidine-based analgesic strategies [3-5]. However, the absence of a non-dexmedetomidine control group means that this audit cannot determine whether low opioid requirements were directly attributable to dexmedetomidine or to other components of multimodal analgesia, regional anaesthesia use or surgical case mix.

This audit has several limitations. It was retrospective, observational, and conducted at a single centre, meaning that causality cannot be established and generalisability may be limited. There was no control group of patients who did not receive dexmedetomidine, which limits interpretation of the generally favourable PACU recovery outcomes and low postoperative opioid requirements, as these findings cannot be attributed directly to dexmedetomidine. No multivariable analysis was performed, so potential confounding by procedure duration, anaesthetic technique, surgical specialty, dexmedetomidine dose, concurrent sedative or analgesic medications, intraoperative fluid therapy, patient comorbidities and case complexity could not be adjusted for. Subgroup analyses were also limited by small sample sizes and low event numbers, particularly in the regional anaesthesia and procedural sedation group. Therefore, the results should be considered descriptive and hypothesis-generating rather than confirmatory. Although PACU clinical recovery time was based on documented discharge readiness rather than actual PACU departure time, discharge readiness was determined retrospectively from routine clinical documentation and may not have been standardised to the same degree as a prospectively applied PACU scoring system.

Despite these limitations, this audit reflects real-world perioperative practice at a tertiary hospital and identifies dexmedetomidine timing as a potentially relevant marker of PACU recovery patterns. The findings should not be used to support a definitive change in dexmedetomidine timing practice, but they provide a basis for further prospective evaluation. Future studies should include larger cohorts, comparison with non-dexmedetomidine controls, and multivariable adjustment for procedure duration, anaesthetic technique, and surgical complexity.

## Conclusions

In this retrospective single-centre quality improvement audit, PACU recovery outcomes were generally favourable in adult surgical patients who received intraoperative dexmedetomidine, and postoperative opioid requirements were low. Dexmedetomidine administration within 60 minutes of procedure completion was associated with shorter PACU clinical recovery time compared with earlier administration; however, this finding should be interpreted cautiously due to the retrospective observational design, absence of a non-dexmedetomidine control group, and potential confounding by procedure duration, anaesthetic technique, dexmedetomidine dose, surgical complexity and case mix. Bradycardia, hypotension and postoperative nausea and vomiting were numerically more common with later administration, but these differences were not statistically significant. These findings are therefore exploratory and hypothesis-generating rather than confirmatory. Further prospective studies with adjusted analyses are required to determine whether dexmedetomidine timing independently influences PACU recovery and early postoperative complications.
